# Alpha lipoic acid attenuates inflammatory response during extracorporeal circulation

**DOI:** 10.5830/CVJA-2013-067

**Published:** 2013-10

**Authors:** Ihsan Sami Uyar, M Besir Akpinar, Veysel Sahin, Abdulhadi, Suleyman Onal, Ibak Gonen, Abdulhadi Cihangir Uguz, Oktay Burma

**Affiliations:** Department of Cardiothoracic Surgery, Faculty of Medicine, Şifa University, İzmir, Turkey; Department of Cardiothoracic Surgery, Faculty of Medicine, Şifa University, İzmir, Turkey; Department of Cardiothoracic Surgery, Faculty of Medicine, Şifa University, İzmir, Turkey; Department of Cardiothoracic Surgery, Faculty of Medicine, Şifa University, İzmir, Turkey; Department of Microbiology, Faculty of Medicine, Suleyman Demirel University, Isparta, Turkey; Department of Infectious Diseases, Faculty of Medicine, Suleyman Demirel University, Isparta, Turkey; Department of Biophysics, Faculty of Medicine, Suleyman Demirel University, Isparta, Turkey; Department of Cardiothoracic Surgery, Faculty of Medicine, Firat University, Elazig, Turkey

**Keywords:** extracorporeal circulation, systemic inflammatory response, oxidative stress, a-lipoic acid

## Abstract

**Aim:**

Extracorporeal circulation (ECC) of blood during cardiopulmonary surgery has been shown to stimulate various pro-inflammatory molecules such as cytokines and chemokines. The biochemical oxidation/reduction pathways of a-lipoic acid suggest that it may have antioxidant properties.

**Methods:**

In this study we aimed to evaluate only patients with coronary heart disease and those planned for coronary artery bypass graft operation. Blood samples were obtained from the patients before the operation (P1) and one (P2), four (P3), 24 (P4) and 48 hours (P5) after administration of a-lipoic acid (LA). The patients were divided into two groups, control and LA treatment group. Levels of interleukin-6 (IL-6) and -8 (IL-8), complement 3 (C3) and 4 (C4), anti-streptolysin (ASO), C-reactive protein (CRP) and haptoglobin were assessed in the blood samples.

**Results:**

Cytokine IL-6 and IL-8 levels were significantly higher after surgery. Compared with the control groups, LA significantly decreased IL-6 and IL-8 levels in a time-dependent manner. CRP levels did not show significant variation in the first three time periods. CRP levels were higher after surgery, especially in the later periods. These results demonstrate that CRP formation depends on cytokine release. C3 and C4 levels were significantly higher after surgery than in the pre-operative period. LA treatment decreased C3 and C4 levels. Therefore, LA administration may be useful for the treatment of diseases and processes where excessive cytokine release could cause oxidative damage.

**Conclusions:**

Our findings suggest a possible benefit of using LA during cardiac surgery to reduce cytokine levels.

## Abstract

Nowadays, extracorporeal circulation (ECC) is commonly employed by surgeons in many cardiac surgical procedures, with the aim of keeping patients’ circulatory parameters at steady levels. ECC is therefore a vital tool for good execution of cardiac surgery.

However, ECC is also associated with some disadvantages. The immune system generates a systemic inflammatory response to the artificial surfaces of the ECC circuit.^1^ ECC-related inflammation can result in adverse effects, including dysfunction of the myocardium, lung, kidney and liver, which may cause multi-organ failure, stroke and significant death rates.^2^-^4^ The molecular mechanism of ECC-induced inflammation needs clarification.^2^

Inflammation is a defensive mechanism of vascularised tissue, which functions as part of the normal host inspection mechanisms to destroy or quarantine both harmful agents and damaged tissue resulting from physiological processes.^5^ As a result of inflammation, levels of different cytokines either rise or fall. These changes in cytokine levels and activation of the complement system are well-known parameters used in the laboratory to determine the inflammatory response.

α-Lipoic acid (LA) is an essential antioxidant that plays a crucial role in the mitochondrial respiratory pathway, including dehydrogenase reactions. It acts with reactive oxygen species (ROS) such as hydroxyl, peroxyl and superoxide radicals and also protects the cellular membrane structure by interacting with glutathione (GSH), which is the preferred substrate of vitamin E.^6^,^7^ The biochemical oxidation/reduction pathways of LA suggest that it may have potent antioxidant properties.

Cardiopulmonary bypass (CPB) -triggered systemic inflammatory response is associated with increased cytokine levels, namely interleukin-6 (IL-6) and interleukin-8 (IL-8). These cytokines may be responsible for many undesirable sequelae associated with CPB.^8^

The main target of our current study was to investigate whether LA administration could modulate the ECC-triggered inflammatory response during ex vivo ECC. Standard procedures were used and 1 200 mg LA was administered into the ECC circuit. The effects of LA on IL-6, IL-8, ASO, CRP and haptoglobulin release were investigated.

## Methods

The study protocol was approved by the ethics committee of the Faculty of Medicine, Firat University, and it adheres to the Declaration of Helsinki. Individuals were informed about the aims, procedures and possible risks of the study and gave informed, written approval.

Inclusion criteria were clinically diagnosed coronary artery disease requiring coronary artery bypass operation (CABG), age between 41 and 70 years and body mass index > 25 kg/m^2^. Thirty patients with coronary artery disease undergoing CABG were included in this study. Patients were randomly divided into two groups, the control and LA treatment group.

Data obtained from 15 patients who were operated on in the standard manner were evaluated in the control group (*n* = 15; eight males, seven females; mean age 63.43 ± 6.12 years, range 41–69 years). During the same dates, another 15 patients who had undergone coronary bypass surgery and received LA in the prime solution of the cardiopulmonary bypass were evaluated within the LA group (*n* = 15; seven males, eight females; mean age 61.16 ± 4.72 years, range 42–70 years).

Blood samples (10 ml) were taken 24 hours before (P1), and one (P2), four (P3), 24 (P4) and 48 hours (P5) after the operation. Five blood samples were taken from each patient by venipuncture. The mean follow-up time for all patients was 24 ± 9.4 months (range 12–48 months). Patients were followed postoperatively after the first month, and then every six months.

Levels of IL-6 and IL-8 were evaluated by enzyme-linked immunosorbent assay (ELISA) using PeliKine compact human ELISA (Amsterdam, Netherlands) kits according to the manufactuter’s instructions. Results are presented in pg/ml. C3 and C4 levels in the serum were analysed nephelometrically with Dade Behring C3 and Dade Behring C4 kits (Marburg, Germany) using a Behring nephelometer 100 (Illinois, USA). Results are presented in g/l.

C-reactive protein (CRP) and anti-streptolysin O (ASO) levels were analysed with the Schiapparelli biosystems (Columbia, USA) turbidimetric method. Results are presented in IU/ml for ASO and mg/l for CRP as described elsewhere.^9^ Haptoglobin levels were evaluated with the Space Schiapparelli Inc (Columbia, USA) turbidimetric method, and results are given in g/l.

## Statistical analysis

Data are expressed as means ± SEM of the numbers of analyses. Statistical significance was analysed using the SPSS program (SPSS, 10.0; Inc, Chicago, IL, USA). To compare the difference between groups, statistical significance was calculated by the Mann–Whitney *U*-test with Spearman rank order correlation test. A *p*-value lower than 0.05 was defined to indicate statistically significant differences.

Since the ECC circuit causes haemodilution, correction was done according to the haematocrit for concentrations of cytokines obtained by the ELISA method. A correction factor for the haematocrit was calculated by dividing the baseline haematocrit by the haematocrit values measured at the sampling time points during ECC.^10^ Values were multiplied by this factor to adjust for haemodilution.

## Results

**Fig. 1.**
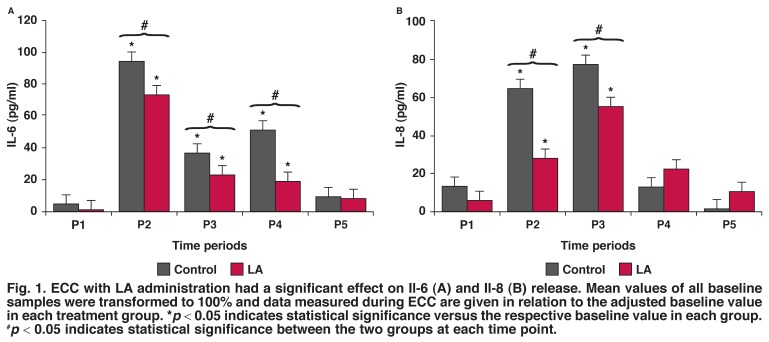
ECC with LA administration had a significant effect on Il-6 (A) and Il-8 (B) release. Mean values of all baseline samples were transformed to 100% and data measured during ECC are given in relation to the adjusted baseline value in each treatment group. *p < 0.05 indicates statistical significance versus the respective baseline value in each group. ^#^*p* < 0.05 indicates statistical significance between the two groups at each time point.

**Fig. 2.**
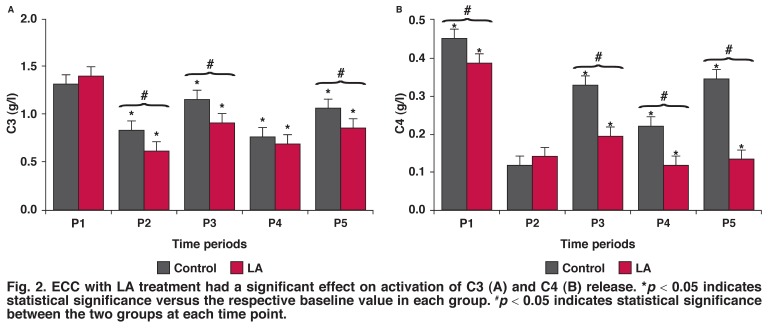
ECC with LA treatment had a significant effect on activation of C3 (A) and C4 (B) release. *p < 0.05 indicates statistical significance versus the respective baseline value in each group. ^#^*p* < 0.05 indicates statistical significance between the two groups at each time point.

**Fig. 3.**
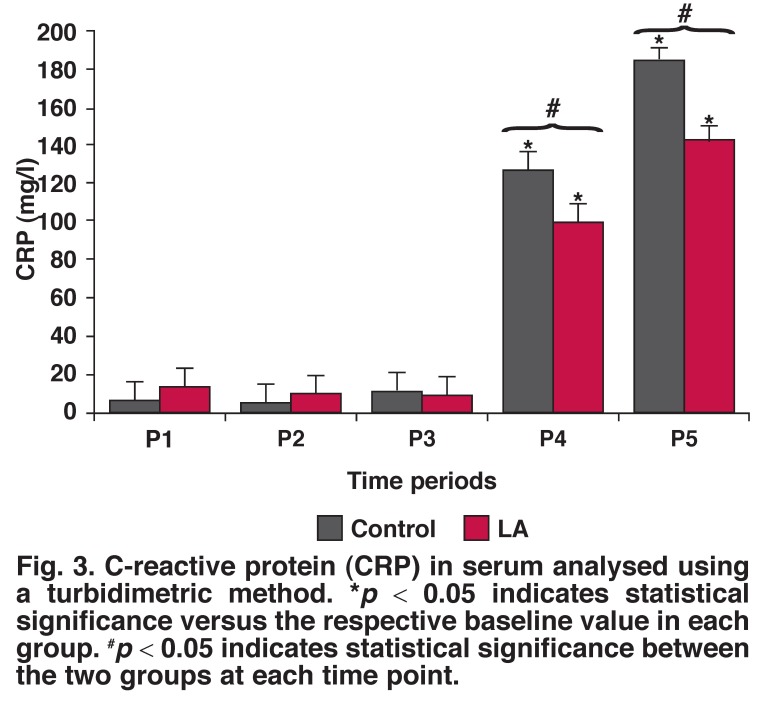
C-reactive protein (CRP) in serum analysed using a turbidimetric method. *p < 0.05 indicates statistical significance versus the respective baseline value in each group. ^#^*p* < 0.05 indicates statistical significance between the two groups at each time point.

**Fig. 4.**
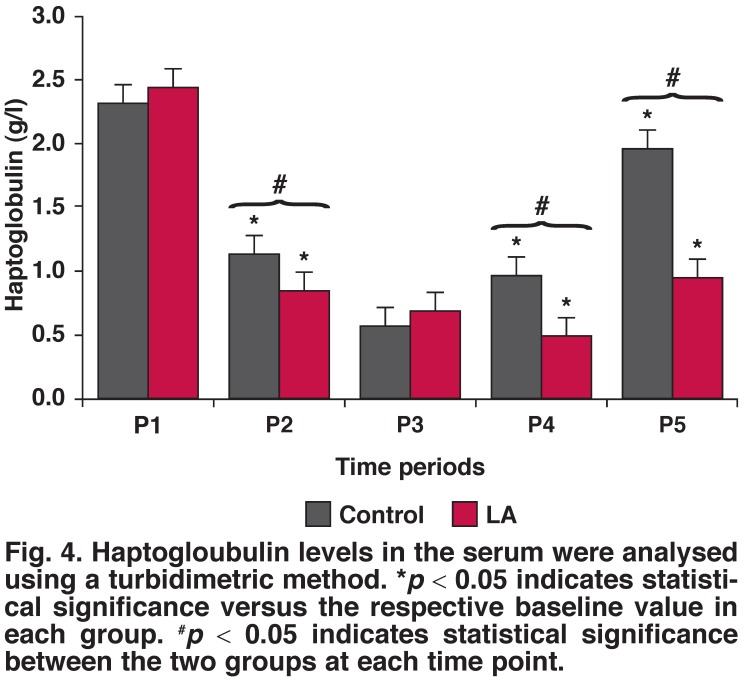
Haptogloubulin levels in the serum were analysed using a turbidimetric method. *p < 0.05 indicates statistical significance versus the respective baseline value in each group. ^#^*p* < 0.05 indicates statistical significance between the two groups at each time point.

**Fig. 5.**
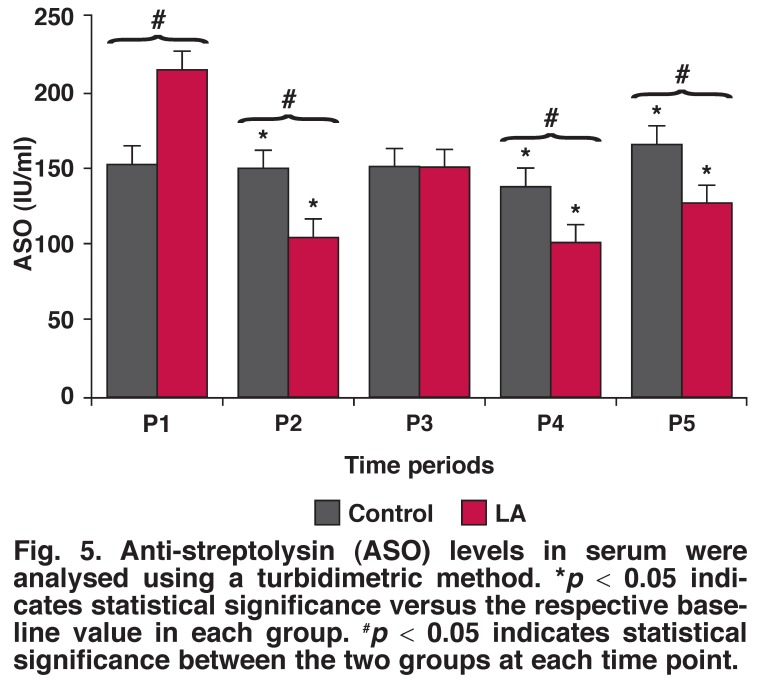
Anti-streptolysin (ASO) levels in serum were analysed using a turbidimetric method. *p < 0.05 indicates statistical significance versus the respective baseline value in each group. ^#^*p* < 0.05 indicates statistical significance between the two groups at each time point.

To examine the effect of LA on synthesis of IL-6, IL-8, C3, C4, ASO, CRP and haptoglobulin, LA was introduced into the ECC and blood samples were drawn before and after CPB at different time periods (Figs 1, 2, 3, 4, 5). As shown in Fig. 1A, IL-6 levels initially increased and then decreased after LA administration. There was a detectable cytokine release after one hour of surgery, with the maximum effect obtained after one hour of ECC (*p* < 0.05). Thereafter IL-6 levels decreased in a time-dependent manner, compared with the controls and samples taken before surgery. Therefore, treatment with LA caused reduced IL-6 levels, compared with controls.

Similarly, LA decreased IL-8 levels significantly (*p* < 0.05), compared with the controls Fig. 1B. IL-8 levels were highest in the P3 period.

C3 levels were high pre-operatively. LA administration decreased C3 levels in the post-operative period compared to P1 Fig. 2A. The effect of LA was clearly seen in C4 levels (Fig. 2B), which were lower in P2, P3, P4, P5 samples than in P1 (*p* < 0.05).

CRP levels did not show any significant change in the first three time periods (Fig. 3). Interleukin release was associated with CRP levels. After interleukins were released, CRP was synthesised and increased significantly in the P4 and P5 periods (*p* < 0.05).

## Discussion

The main purpose of the current study was to investigate the preventive effects of LA on ECC-triggered inflammatory events. We systematically investigated the generation of pro-inflammatory cytokines IL-6 and IL-8, and ASO, CRP and haptoglobulin after ECC. Recent studies on inflammatory reactions occurring during and after CPB have improved our understanding of the contribution of inflammatory pathways to disease.

Our results clearly demonstrate that ECC triggered a pro-inflammatory cytokine release during CPB, which was significantly inhibited by LA administration into the ECC circuit. Warren *et al.* found that contact of the patient’s blood with the artificial surfaces of the ECC circuit triggered a systemic inflammatory response related to increased secretion of IL-1β, IL-6 and IL-8.^4^

The inflammatory response is associated with the production of reactive oxygen species (ROS). The primary source of ROS during ECC is thought to be neutrophil granulocytes,^11^ which also release enzymes. ECC activates neutrophil granulocytes,^12^ which then trigger an inflammatory response with complement activation after cytokine release. This may stimulate further cardiac injury.^13^ The activation of neutrophil granulocytes may occur following complement activation by both immunological or non-immunological (heparin-protamine, endotoxin) pathways.^14^ Up-regulation of adhesion molecules may be stimulated by cytokines on the cardiac cells, which allow neutrophil granulocytes to discharge ROS products.^15^

More recently, Salinthone *et al.* have shown that LA displays a non-redox anti-inflammatory role^16^ by moderating a diverse range of signaling cascades, which mediate these processes. Moreover, LA induces the production of the immunomodulator cAMP in human inflammatory cells by activating the prostaglandin E2 (PGE2), EP2 and EP4 receptors.^17^ In addition, in Wang and co-workers’ ECC model for CPB, the myocardium produced inflammatory mediators and ROS during ischaemia–reperfusion, which would contribute to cardiac functional reduction and apoptosis.^18^

Similarly, Sawa *et al.* reported that cardiac myocytes exposed to ischaemia–reperfusion were shown to produce IL-6 in several experimental models.^19^ In order to improve clinical outcomes in open-heart surgery with CPB, oxidative stress should be prevented by decreasing reperfusion injury and inflammation.

Despite improvements in surgical techniques, inflammation continues to be an important problem during these procedures. Wan *et al.* have shown that CPB may induce complement or leukocyte activation, endotoxin release, the expression of adhesion molecules, and the release of inflammatory mediators.^2^ Moreover, the heart itself is a major source of inflammatory mediators and oxygen-derived free radical species that are likely to contribute to the impairment of cardiac pump function.^20^

LA administration has been shown to be advantageous in a number of oxidative stress models such as ischaemia–reperfusion injury, diabetes and cataract formation. In the present study, the beneficial effects of LA were manifested by statistically significant decreases in plasma IL-6, IL-8, C3, C4, CRP and haptoglobulin levels. LA decreased levels of IL-6 in the P2, P3 and P4 periods and decreased levels of IL-8 in the P2 and P3 periods. When compared with the controls, LA significantly decreased IL-6 and IL-8 synthesis in a time-depended manner. LA may act as extra- and intracellular redox signaling couples and a powerful free radical scavenger, suggesting that LA has a possible therapeutic agent in surgeries where ECC is used.^21^

Similarly, Steinberg *et al.* reported that IL-6 levels increased after protamine administration and reached a maximum level three hours after bypass. At 24 hours after bypass, IL-6 levels remained above the levels measured at initiation. Our results showed that IL-6 levels in both groups were above the P1 levels at P2, P3 and P4. It has been previously reported that LA was able to increase intracellular GSH levels, which is the most abundant cellular antioxidant, by acting as a buffer system for the thiol redox state.^6^,^22^ Nowadays, there is strong evidence that LA is one of the modifiers of critical protein thiolates and therefore may influence the pathways of thiol redox state.^23^,^24^

Furthermore, GSH is implicated in the recycling of antioxidant vitamins such as vitamins E and C, which participate in modulating the activity of superoxide dismutase enzyme. Currently, there is mounting evidence that LA increases the levels of the cellular antioxidant enzyme GSH by acting as a transcriptional inducer of genes governing GSH synthesis.^25^ Glutathione peroxidase (GSH-Px) and GSH act as antioxidant molecules and have protective effects against reactive oxygen-derived molecule-triggered degeneration. GSH is one of the most important antioxidant molecules for removing lipid hydroperoxides and hydrogen peroxide.^26^,^27^ It is one of the precursors for catalysing hydrogen peroxide to water.

The two major sources of intracellular ROS production are mitochondria and the plasma membrane-bound multicomponent enzyme complex NADPH oxidase.^28^ Kagan *et al.* also mentioned that LA interacts with NADPH or NADH-dependent electron transport chains to recycle vitamin E.^29^ LA is well known as an inhibitor of nuclear factor (NF-kβ).^7^ LA decreases TNF-α-induced NF-kβ activation and the expression of adhesion molecules in endothelial cells, and thereby it may reduce the inflammatory response.^25^,^30^,^31^

In inflammatory diseases, membrane damage appears frequently in cells that incite lipid peroxidation and disturbances in membrane structure.^32^ When lipid peroxides aggregate to a certain level, they leak from the cells into the blood and increase lipid peroxidation in the blood plasma. Melek *et al.* determined increased levels of CRP during ECC.^33^ CRP is one of the indicators of inflammation activated by cytokines in the liver.

In our study, the levels of CRP increased in the P4 period, following IL-6 and IL-8 increase. This demonstrated that CRP activation is depended on LA synthesis. These changes are also considered to be a consequence of imbalance between oxidant products and antioxidant defense mechanisms. This kind of systemic inflammatory response to CPB has the potential of bringing about clinical and cellular disorders.

Maulik *et al.*^34^ demonstrated that oxidative stress triggered apoptosis in re-perfused hearts in swine. This relatively unknown anti-inflammatory effect of LA may contribute to the inhibition of ECC-induced inflammation in vivo and reduce ECC-related adverse effects.

## Conclusion

ECC is an important innovation in CPB, but its safety is not guaranteed due to the inflammatory reaction generated by ECC.^35^,^36^ Systemic inflammatory reactions cause serious complications, which may affect postoperative mortality in cardiac surgery patients. Therefore the originality of our findings and the potential benefits of using LA during cardiac surgery could be useful.^37^,^38^ Future research will be directed at finding the unique pharmacological and biological agents or their combinations, which may effectively reduce ECC-caused inflammatory responses. An appropriate strategy to inhibit ECC-triggered inflammation could be beneficial for patients undergoing cardiac surgery using ECC.
